# Study of auditory pathways in type 1 diabetes mellitus through brainstem auditory evoked potentials and contralateral acoustic reflex

**DOI:** 10.1590/2317-1782/20232021022

**Published:** 2023-05-08

**Authors:** Luciene da Cruz Fernandes, Caio Leônidas Oliveira de Andrade, Luis Fernando Fernandes Adan, Ana Marice Teixeira Ladeia

**Affiliations:** 1 Departamento de Fonoaudiologia, Instituto Multidisciplinar de Reabilitação e Saúde, Universidade Federal da Bahia – UFBA - Salvador (BA), Brasil.; 2 Departamento de Ciências da Saúde, Universidade do Estado da Bahia – UNEB - Salvador (BA), Brasil.; 3 Departamento de Pediatria, Faculdade de Medicina, Universidade Federal da Bahia – UFBA - Salvador (BA), Brasil.; 4 Pós-graduação em Medicina e Saúde, Escola Bahiana de Medicina e Saúde Pública – EBMSP - Salvador (BA), Brasil.

**Keywords:** Diabetes Mellitus Type 1, Glucose Metabolism Disorders, Reflex Acoustic, Evoked, Potentials, Auditory, Brain Stem, Hearing, Diabetes Mellitus Tipo 1, Transtornos do Metabolismo da Glicose, Reflexo Acústico, Potenciais Evocados Auditivos do Tronco Encefálico, Audição

## Abstract

**Purpose:**

To investigate the functionalities of the neural pathways through the auditory evoked potentials of the brainstem and the contralateral stapedial acoustic reflexes in normal-hearing individuals with type 1 diabetes mellitus, in order to detect possible alterations in the central auditory pathways.

**Methods:**

This is a cross-sectional study with a comparison group and a convenience sample, consisting of 32 individuals with type 1 diabetes mellitus and 20 controls without the disease. All subjects had hearing thresholds within normal limits and type A tympanometric curves. The acoustic reflex arc and brainstem auditory potentials were investigated. Statistical analyses were performed using the SPSS 17.0. The Chi-square test, Student´s t-test, and Multiple linear regression were used.

**Results:**

The auditory thresholds of the acoustic reflex were statistically lower in the group with the disease at frequencies of 0.5 kHz and 1.0 kHz in the left ear (p=0.01 and p=0.01, respectively). The absolute latencies III and V of the auditory potentials of the brainstem in the right ear and V in the left ear were increased in subjects with type 1 diabetes mellitus (p=0.03, p=0.02 and p=0.03, respectively).

**Conclusion:**

The findings suggest that subjects with type 1 diabetes mellitus are more likely to present alterations in the central auditory pathways, even with auditory thresholds within normal limits.

## RESUMO

**Objetivo:**

Investigar a funcionalidade das vias neurais por meio dos potenciais evocados auditivos de tronco encefálico e os reflexos acústicos estapedianos contralaterais em sujeitos com diabetes *mellitus* tipo 1 normo-ouvintes, a fim de detectar possíveis alterações nas vias auditivas centrais.

**Método:**

Trata-se de um estudo transversal com grupo de comparação, e amostra de conveniência, composta por 32 sujeitos com diabetes mellitus tipo 1 e 20 controles sem a doença. Todos os sujeitos apresentavam limiares auditivos dentro dos padrões de normalidade e curva timpanométrica tipo A. Foram investigados o arco-reflexo acústico e os potenciais auditivos de tronco encefálico. As análises dos resultados foram realizadas no SPSS 17.0. Utilizou-se o Teste Qui Quadrado, Teste T de Studant e Regressão linear múltipla.

**Resultados:**

Os limiares auditivos do reflexo acústico foram estatisticamente menores no grupo com a doença nas frequências de 0,5 kHz e 1,0 kHz na orelha esquerda (p=0,01 e p=0,01, respectivamente). As latências absolutas III e V dos potenciais auditivos de tronco encefálico da orelha direita e V da orelha esquerda estavam aumentadas em sujeitos com diabetes mellitus tipo 1 (p=0,03, p=0.02 e p=0,03, respectivamente).

**Conclusão:**

Os achados sugerem que sujeitos com diabetes mellitus tipo 1 estão mais propensos a apresentar alterações nas vias auditivas centrais, mesmo com limiares auditivos dentro dos padrões de normalidade.

## INTRODUCTION

Type 1 diabetes mellitus (T1DM) is a multifactorial disease, the environmental, genetic, and immunological factors of which can interact. These complex interactions culminate in the autoimmune reaction that damages the pancreatic beta cells, causing a complete insulin deficiency in the body^(1)^. It is estimated that the global T1DM prevalence rate is approximately 5% to 10%^(2)^.

The association between diabetes mellitus and impaired auditory pathways remains contentious amongst researchers, as studies suggest the frequency of hearing loss in diabetic patients can range anywhere between 5,17% and 48%^(3,4)^.

The proposed etiopathogenic mechanisms are associated with chronic complications of the disease. These include microangiopathy of the capillaries of the inner ear and neuropaty^(3)^, besides premature metabolic complications such as non-enzymatic glycation related to the hyperactivity of free oxygen radicals^(5)^. Thus, it is possible that for diabetes mellitus, the hearing loss may have endocochlear, retrocochlear, or combined sites of origin^(6)^.

The audiometric test does not adequately assess the structures most vulnerable to injuries in individuals with T1DM; therefore, it does not present a method capable of detecting subclinical abnormalities. There is a consensus in the literature about the factors that promote clinical complications, attesting that the most affected site is located predominately in the central nervous system, secondary to inadequate metabolic control and early start of the disease in this population. Studies indicate that irregular metabolic control has potentially deleterious effects on the auditory pathways and may contribute to the onset of peripheral auditory disorders^(7,8)^ as well as injury to the central auditory structures, as evidenced by an increase in absolute latencies when the auditory brainstem evoked potentials were analyzed^(9,10)^.

Given the above, it is not clear in the literature whether type 1 diabetes mellitus can affect the central auditory pathways early. The results are still controversial and based on different clinical designs, with heterogeneous samples, with much of the information in the literature based on older patients and many already with other associated comorbidities. Thus, it makes it difficult to understand the real pathophysiological processes of DM1 in hearing at early ages of development, which would be essential for monitoring associated comorbidities and better quality of life for these patients.

Therefore, This study aimed to investigate the neural synchrony of the auditory pathway in T1DM with hearing thresholds within the normal range through brainstem auditory evoked potentials (BAEPs) and contralateral acoustic reflex thresholds to detect possible hearing loss in the absence of psychoacoustic alteration, thereby confirming the presence of subclinical changes in this population.

## METHODS

This is a cross-sectional study with a comparison group comprised by a sample of outpatients, which included 32 T1DM patients and 20 nondiabetic controls. In the T1DM group, 56.3% of the patients were male, with a mean age of 21.0 ± 6.1 years, and the average duration of the disease was 8.0 ± 1.7 years. The experimental group comprised all patients who sought care at the Public and Reference Center (PRC, Salvador, Bahia, Brazil) for the treatment of diabetes mellitus. Eligibility included confirmed diagnosis of T1DM through the patient’s clinical history and fasting blood glucose test. Additionally, the participants required audiometry results with hearing thresholds ≤20 dB HL and normal tympanometry.

The control group, which comprised individuals who frequented a popular pharmacy near PRC, had 56.0% male participants, with a mean age of 22.7 ± 7.6 years, presenting with normal capillary blood glucose levels. The exclusion criteria were as follows: a history of neurological or psychiatric disease, history of adverse hearing conditions, disabling motor difficulties, other forms of metabolic diseases other than T1DM to avoid associated comorbidities^(2)^, such as hearing loss detected in the threshold tonal audiometry and immittanciometry.

### Laboratory data

With regard to the capillary fasting blood glucose levels (VR≤101 mg/dL), the average of the last three results recorded in the participant’s medical records was used, along with the glycated hemoglobin (HbA1c) levels (VR=3.7%–6.5%) recorded over the previous three months^(2)^.

### Audiological evaluation

The audiological evaluation was divided into two parts. The first one sought to exclude or include individuals from the study’s sample. In this part, all the participants were subjected to the following audiological evaluations. a) Otoscopy was performed to rule out the presence of cerumen or foreign objects that could interfere with obtaining correct audiometric and tympanometric results. b) Audiometry was performed in a sound-treated booth using a Danplex DA65 audiometer (Danplex, Denmark), with supra-aural TDH-39 headphones. Air-conduction thresholds at 0.25 kHz, 0.5 kHz, 1 kHz, 2 kHz, 3 kHz, 4 kHz, 6 kHz, and 8 kHz were obtained in both ears, followed by determination of the speech recognition threshold (SRT) and speech discrimination index (SDI), performed in an acoustically-treated cabin. Individuals with thresholds up to 20 dBHL at all tested frequencies were considered c) Immittance tests were conducted to determine the tympanogram (tympanometry using 226-Hz probe tone) considered normal when classified in type A curve^(11)^ and contralateral acoustic reflex thresholds at 0.5 Hz, 1 Hz, and 2 kHz present at normal levels^(12,13)^.

BAEP testing was performed using the Interacoustics EP25 device (Interacoustics Co., Assens, Denmark). The stimuli were broadband clicks presented at one intensity (80 dB HL), two polarities (rarefaction, condensation), and one rate (30 clicks/second) in each ear, monaurally. The BAEP was recorded differentially using a gold surface electrode secured to the ipsilateral mastoid for the active (inverting) channel. A surface electrode attached to the high forehead (Fz) served as the reference (noninverting) electrode, and a surface electrode attached to the brow (Fpz) served as the ground. Efforts were made to maintain electrode impedances below 3 Ω. The recorded signal was amplified (100,000 times), filtered (0.3–3.0 kHz), and digitized for subsequent analyses. The BAEPs were collected as two samples of 2000 runs for each stimulus polarity. The averages of the two samples were compared to determine whether BAEP morphology was replicable. Adopting as normality criteria recommended by Hood^(14)^.

The research was approved by the Committee of Ethics in Research of CEDEBA and the approval number is 13.11.09. The patients were informed about the procedures, and those involved signed the Free and Informed Consent Form.

### Statistical analyses

Statistical analyses were performed using the SPSS software (Statistical Package for Social Sciences) version 17.0 (IBM Corp., Chicago, IL, USA). The chi-square test was adopted to analyze the association between qualitative variables. The Student´s t-test was used to verify the comparison between the average of two groups. Multiple linear regression was used to verify relationships between age, age at diagnosis, time since diagnosis, and hearing thresholds with BAEPs and acoustic reflex thresholds.

## RESULTS

In the group T1DM, 56.3% subjects were male, with a mean age of 21.0 + 6.1 years. The average patient age at diagnosis was 9.0 + 4.1 years, and average time since diagnosis was 8.0 + 1.7 years. In the control group, 56.0% were male, with a mean age of 22.7 ± 7.6 years. There was no statistically significant difference between the study and control groups concerning age and sex (p > 0.05).

Tonal hearing threshold frequencies were 4 kHz in the right ear (p<0.01) and 2 kHz (p=0.04) and 4 kHz (p=0.04) in the left ear. The hearing threshold in the T1DM group was elevated, compared with the results for the same in the control group (Table 1).

**Table 1:** Comparison of mean hearing thresholds between Type 1 diabetes mellitus (T1DM) group and control group

	Right ear (dBHL)			Left ear (dBHL)		
Frequencies(Hz)	Control Group	T1DM Group	p	Control Group	T1DM Group	p
	(n=20)	(n=32)		(n=20)	(n=32)	
250	15.00 (±5.77)	11.87 (±5,64)	0.34	11.25 (±7.50)	10.62 (±5.78)	0.94
500	10.00 (±9.12)	11.87 (±5.04)	0.69	10.00 (±5.77)	10.31 (±4.91)	0.91
1000	5.00 (±5.00)	7.96 (±5.05)	0.20	0.375 (±2.50)	7.65 (±6.21)	0.18
2000	5.00 (±5.77)	9.69 (±6.71)	0.23	-1.25 (±4.78)	0.797 (±9.91)	0.04*
3000	2.50 (±8.66)	11.87 (±9.04)	0.06	0.00 (±7.07)	11.40 (±10.41)	0.21
4000	2.50 (±5.00)	14.84 (±9.37)	<0.01*	2.50 (±6.45)	14.21 (±13.56)	0.04*
6000	0.37 (±10.30)	13.12 (±9.22)	0.09	8.75 (±6.29)	13.59 (±11.44)	0.43
8000	0.25 (±6.45)	8.12 (±8.00)	0.16	3.75 (±4.79)	8.59 (±9.61)	0.34

* Statistically significant (Student 's T test)

Values represent the mean ± standart deviation

**Legend:** T1DM: Type 1 Diabetes Mellitus; dBHL: Decibel Level Hearing; Hz: Hertz

In the group T1DM, 84% of individuals had inadequate metabolic control of capillary blood glucose (77% hyperglycemia and 7% hypoglycemia) and 90.0% had higher levels of glycated hemoglobin. All subjects in the control group had normal glucose levels.

As regards the association with glycemic control, the tonal hearing thresholds at frequencies of 8 kHz were worse in the subjects T1DM without glycemic control [10.86(±10.18) vs. 1.25(±2.5), p=0,04], suggesting worse hearing in this subgroup. No statistically significant difference was observed between the tonal hearing thresholds in the T1DM and those without adequate control of their HbA1c.

In the T1DM group there was a statistically significant correlation between tonal hearing thresholds and age at the frequencies of 3 kHz (r = 0.43, p = 0.02) and 4 kHz (r = 0.45, p = 0.02) in the right ear and 6 kHz (r = 0.42, p = 0.02) in the left ear. There was also an association between tonal hearing thresholds and patient´s age at diagnosis at the frequencies of 3 kHz (r = 0.46, p = 0.01) and 4 kHz (r = 0.44, p = 0.02) in the right ear and 6 kHz (r = 0.47, p = 0.01) in the left ear.

The higher the age of the patient at diagnosis, the worse the tonal hearing threshold, i.e. the higher the level of hearing impairment. No statistically significant association between the tonal hearing thresholds and the time elapsed since diagnosis was observed.

In the T1DM group, acoustic reflexes thresholds were elicited using lower intensity sounds at the frequencies of 0.5 and 1 kHz in the left ear. (Table 2).

**Table 2:** Comparison of mean contralateral acoustic reflex between Type 1 diabetes mellitus (T1DM) group and control group

	Right ear (dBHL)				Left ear (dBHL)		
Frequencies (Hz)	Control Group	T1DM Group	p	Control Group		T1DM Group	p
	(n=20)	(n=32)		(n=20)		(n=32)	
500	93.75 (±4.78)	88.33(±8.57)	0.25	102.50 (±5.00)		90.00(±4.85)	<0.01*
1000	93.75 (±7.50)	87.22(±6.47)	0.12	100.00 (±8.16)		87.78(±5.48)	0.01
2000	90.00 (±7.07)	85.28(±6.52)	0.26	91.25 (±10.31)		83.61(±5.08)	0.17

* Statistically significant (Student's T test)

Values represent the mean ± standart deviation

**Legend:** T1DM: Type 1 Diabetes Mellitus; dBHL: Decibel Level Hearing; Hz: Hertz

Similarly, the acoustic reflexes thresholds were elicited by lower intensity sounds at the frequency of 0.5 kHz in the left ear in the subgroup of individuals T1DM without control of their HbA1c (89.50±3.68 vs. 97.50±3.53, p=0.04).

With regard to capillary blood glucose, there was no association observed between this variable and the contralateral acoustic reflexes thresholds in the group T1DM. Moreover, the association between the values of contralateral acoustic reflexes thresholds with sex, age or tonal hearing threshold in the group T1DM was not statistically significant.

With respect to the patient´s age at diagnosis, there was a positive correlation between contralateral acoustic reflexes thresholds at the frequency of 0.5 kHz in the right ear (r = 0.60, p = 0.02). However, in the control group, an inverse correlation was observed between contralateral acoustic reflexes thresholds at the frequency of 0.5 kHz (r = -0.73, p = 0.04) and 2000 Hz (r = -0.69, p = 0.05) in the left ear. The older the patient was, the lower the intensity needed to elicit acoustic reflexes.

Table 3 shows that the absolute latencies of waves III and V in the right ear and V in the left ear were prolonged in the T1DM group in comparison with in the group without the disease.

**Table 3:** Comparison of Mean Auditory Brainstem Response between Type 1 diabetes mellitus (T1DM) group and control group

	Ear Right			Ear Left		
Latencies (ms)	Control Group	T1DM Group	p	Control Group	T1DM Group	p
	(n=20)	(n=32)		(n=20)	(n=32)	
Absolut						
I	1.39 (±0.12)	1.48 (±0.10)	0.08	1.34 (±0.10)	1.40 (±0.12)	0.14
III	3.43 (±0.17)	3.59 (±0.18)	0.03*	3.44 (±0.18)	3.57 (±0.18)	0.10
V	5.36 (±0.16)	5.55 (±0.25)	0.02*	5.39 (±0.11)	5.54 (±0.23)	0.03*
Interpeak						
I-III	2.03 (±0.14)	2.11 (±0.19)	0.14	2.09 (±0.13)	2.16 (±0.17)	0.13
III-V	1.92 (±0.17)	1.96 (±0.20)	0.41	1.94 (±0.21)	1.96 (±0.16)	0.62
I-V	3.96 (±0.16)	4.07 (±0.24)	0.13	4.05 (±0.13)	4.14 (±0.24)	0.12

* Statistically significant (Student's T test)

Values represent the mean ± standart deviation

**Legend:** T1DM: Type 1 Diabetes Mellitus; ms: Millisecond

There was no association observed between the nerve conduction times in the auditory pathway (represented by the I-III, III-V and I-V interpeak intervals), capillary blood glucose, and HbA1c values (normal < 7.0% and elevated ≥ 7.0%) in the group with T1DM. Likewise, no association was found between the results of the BAEP and the patient´s age at diagnosis. Whereas, the time since the diagnosis was associated with the absolute latency of wave V for both the right ear (r = 0.58, p = 0.05) and the left ear (r = 0.72, p = 0.01). No association was observed between BAEP and age or tonal hearing threshold in the group T1DM or the control group.

## DISCUSSION

The present study highlights the possible subclinical hearing alterations in T1DM patients.

Most of the participants presented inadequate metabolic control and clinically harmful conditions to homeostasis in the auditory system. The hypoglycemia and hyperglycemia may change the normal function^(15)^, however, hyperglycemia is the condition most directly related with the appearance of several morphological alterations in the auditory system^(16)^. It is also involved in the decrease of the Na^+^-K^+^-ATPase pump activity, causing labyrinthine hydrops by hyperosmolarity secondary to the accumulation of sodium in the endolymph^(5,17)^.

This consequence can be seen in the difference between the hearing thresholds of individuals with and without T1DM; it can be seen that the group with T1DM presented higher hearing thresholds, despite being within the normal standards. When analyzing only the group with T1DM, it was found that the hearing thresholds were worse when there was inadequate glycemic control. Thus, T1DM may compromise the physiology of the inner ear at a subclinical level. In a previous literature review study, we investigated otoacoustic emissions, and a reduced amplitude was seen in patients with T1DM^(18)^. Histopathological findings of the inner ear of individuals with T1DM also favor this hypothesis, because they demonstrate the thickening of the capillary walls of the *stria vascularis*, the presence of hemorrhage and endolymphatic hydrops, a decreased number of fibers of the spiral blade, degenerative changes in the organ of Corti, and a reduction in external hair cells^(3)^.

However, the present study did not aim to describe alterations in the tonal auditory threshold, but the mean differences between the analyzed, it was verified that the high frequencies were more affected by the disease, particularly in conditions of inadequate metabolic control, corroborating with the existing data in the literature^(19)^. It also showed a worsening hearing prognosis with an increase in age, without following a similar standard for presbycusis^(3)^.

It should be noted that this study found a reduced level of contralateral acoustic reflex thresholds, independent of hearing thresholds, between individuals with and without T1DM. To the best of our knowledge, this is the first study that has shown this result. Others have not found differences in the thresholds of the contralateral acoustic reflex in young T1DM patients^(20)^ or an increase in the acoustic reflex of individuals with T1DM^(21)^.

This reduction in the contralateral reflexes could be explained, initially, as secondary to the notorious hyperglycemic conditions in the sample, considering that this clinical condition decreases the hearing sensitivity due to intracellular hyperosmolarity^17^, which leads to a decrease of the endocochlear potential^(22)^, affecting the eliciting reflex threshold. It can also be due to a probable presence of musculoskeletal alterations of the stapedial muscle secondary to diabetic microangiopathy^(6,16)^ located in the middle and inner ear.

Other morphological alterations such as injury to the motor nerves, myelin degeneration, and damage to axons contribute to the knowledge of the absence and/or reduction of the acoustic reflexes in this population, showing that auditory damages in diabetes may occur in isolated or combined sites of the auditory system^(6)^. In the auditory system, these neural changes can be verified through the inoperability of the efferent system, since in diabetic individuals in situations of hyperglycemia, there is a high bioelectrical activity of the cochlear amplifier, recorded through the increase in the amplitude of otoacoustic emissions, which makes the auditory system more sensitive to sound during decompensation of these metabolic alterations^(23)^.

The BAEP analysis in the present study ratified the endocochlear and/or retrocochlear alterations, which can be seen as an extension of the absolute latencies of waves III and V in T1DM compared with the control group. The increase in absolute latencies in the analysis of BAEP has also been observed in other studies^(6,24,25)^. The increase in the absolute latency of wave V had a positive correlation with the time since the diagnosis, which was not observed in other studies^(10)^.

These studies were conducted in developed countries, where differences in the healthcare systems and public awareness may favor greater effectiveness of glycemic control compared with the population in this study. The population in this study is financially challenged and has poor access to healthcare, such as difficulties in making appointments and obtaining proper medication, among others, leading to a poor metabolic control. Furthermore, with inadequate metabolic control, chronic complications tend to worsen over time.

Acar et al.^(26)^, in BAEP testing, indicated that in pediatric cases of type T1DM, the increase in latencies is indicative of retrocochlear alterations. According to Wu et al.^(27)^, wave III is changed by many structures in the brainstem; thus, it is possible to suggest medial superior olivary complex damage in diabetic individuals. Other studies have determined that there may be changes in the medial olivocochlear efferent neurons in diabetic patients^9,28^ and diabetic rats^27^, particularly those without hearing loss.

Considering the aforementioned findings, the present study, besides contributing toward the basic understanding of the pathophysiology of diabetes mellitus in the auditory system, highlights some clinical implications that attest the need for early intervention, as well as the development of a methodology to monitor the hearing health of this population, on pointing out the existence of subclinical alteration signs in T1DM patients. Furthermore, this study’s limitation in comparing similar findings in subject-specific literature emphasizes that there is a shortage of studies in this field, such as a variety of studies with different protocols and evaluation methods, which is required for the normalization of the included parameters.

## CONCLUSION

The results of this study suggest that individuals with T1DM present a predisposition to the development of hearing loss, particularly in a subclinical manner, observed in the study of brainstem auditory evoked potentials and contralateral acoustic reflex, without presenting specific sites of injury in the auditory pathways, although the central pathways emerge as the most susceptible.
